# Disentangling the importance of microbiological and physico-chemical properties of Ethiopian field soils for the *Striga* seed bank and sorghum infestation

**DOI:** 10.1186/s40793-026-00926-3

**Published:** 2026-07-13

**Authors:** Tamera Taylor, Getahun Benti, Marcio F.A. Leite, Luisa M. Arias-Giraldo, Desalegn W. Etalo, Sewunet Abera, Lorenzo Lombard, Jose G. Maciá-Vicente, Stefan Sanow, Dominika Rybka, Thomas Mostert, Einar Martinez de la Parte, Daniel Legesse, Urgesa Tsega Tulu, Jiregna Daksa, Roy van Doorn, Raycenne Rosa Leite, Taye Tessema, Pedro W. Crous, Dorota Kawa, Eiko E. Kuramae, Jos M. Raaijmakers, Siobhan M. Brady

**Affiliations:** 1 Department of Plant Biology and Genome Center, University of California, Davis, Davis, CA 95616 USA; 2 Department of Microbial Ecology, Netherlands Institute of Ecology (NIOO -KNAW), Droevendaalsesteeg 10, 6708 PB Wageningen, The Netherlands; 3 Institute of Biology, Leiden University, 2333 BE Leiden, The Netherlands; 4 Ethiopian Institute of Agricultural Research, Addis Ababa, Ethiopia; 5 Westerdijk Fungal Biodiversity Institute (WI-KNAW), Uppsalalaan 8, 3584 CT Utrecht, The Netherlands; 6 Plant Hormone Biology Group, Swammerdam Institute of Life Science, University of Amsterdam, Amsterdam, Netherlands; 7 Laboratory of Phytopathology, Wageningen University, Wageningen, The Netherlands; 8 Multidisciplinary Institute for Environmental Studies Ramon Margalef, University of Alicante, Alicante, Spain; 9 Addis Ababa University, Addis Ababa, Ethiopia; 10 Experimental and Computational Plant Development, Utrecht University, Padualaan 8, 10, 3584 CH Utrecht, The Netherlands; 11 Plant Stress Resilience, Utrecht University, Padualaan 8, 10, 3584 CH Utrecht, The Netherlands; 12 Ecology and Biodiversity, Institute of Environmental Biology, Utrecht University, Padualaan 8, 10, 3584 CH Utrecht, The Netherlands; 13 Howard Hughes Medical Institute, University of California, Davis, Davis, CA 95616 USA

**Keywords:** *Striga hermonthica*, *Sorghum bicolor*, sub-Saharan Africa, soil microbiome, chemical analysis, Shiny application

## Abstract

**Background:**

*Striga hermonthica* (*Striga*) is a parasitic weed that severely affects sorghum yield in sub-Saharan Africa. Recent studies highlighted the soil microbiome’s potential to suppress *Striga* through interference with specific stages in its life cycle.

**Results:**

Statistical analyses of data collected from 48 Ethiopian sorghum field soils sampled across a > 1000-km-transect revealed that microbial communities and their interactions with soil physico-chemical properties correlated with *Striga* occurrence in the field. *Striga* infestation of sorghum and seedbank levels were negatively correlated with potassium and sulfur soil content and positively correlated with calcium and magnesium nutrient profile proportions. Microbiome analyses indicated that fungal communities were more responsive than bacteria to changes in *Striga* infestation and seedbank levels, with distinct microbial composition even in soils where *Striga* was not detected. Specific fungal and bacterial genera showed both positive and negative correlations with *Striga* measures, but patterns rarely held across taxonomic levels. To begin to validate these correlations, we tested an isolate from the fungal genus *Neocosmospora*, which negatively correlated with the *Striga* seedbank, and showed that this isolate promotes *Striga* seed germination in vitro. The data and analysis methods are integrated and shared in a public Shiny App for broader analysis and continued research on soil-*Striga* interactions.

**Conclusions:**

This study highlights the complexity of soil-microbiome-*Striga* interactions and the potential for observational studies to reveal candidates for biological control of *Striga*.

**Supplementary Information:**

The online version contains supplementary material available at 10.1186/s40793-026-00926-3.

## Background

*Striga hermonthica* (*Striga*), or witchweed, is a devastating agricultural weed of cereal and legume crops worldwide [1, 2]. For resource-poor farming communities in sub-Saharan Africa, *Striga* causes substantial yield losses of *Sorghum bicolor*, a staple crop used for food, feed, fiber and bioenergy. For sorghum, millet, and rice, the estimated annual production losses due to *Striga* alone exceed 6 million tons of grain. Sorghum exudes strigolactones to recruit arbuscular mycorrhizal fungi (AMF), but this signal molecule is hijacked by *Striga* for seed germination [3, 4]. Upon perception of other host-derived exudates, the *Striga* radicle forms a haustorium that penetrates the host root. Parasitism is complete once *Striga* forms a xylem-xylem connection with its host, through which *Striga* extracts essential nutrients and water. Once *Striga* emerges above ground, it can photosynthesize and complete its life cycle by producing tiny seeds, which are easily dispersed in the topsoil [5, 6]. *Striga*’s high fecundity, exceptional seed dormancy, easy seed dispersal, tight linkage to its host physiology, and a lack of consistently effective management strategies pose an incredible agricultural challenge in *Striga* endemic areas.

Several approaches have been developed that can partially suppress *Striga* parasitism of sorghum. These include resistance breeding [1, 7, 8], chemical approaches (e.g. herbicides) [9, 10] and farming practices (e.g., push-pull, intercropping) [11, 12]. These approaches are not singularly effective, or necessarily financially accessible, nor is resistance breeding easily applicable to local, preferred crop varieties. Hence, an integrated management strategy is needed. In the past decade, interest in the functional potential of the soil microbiome emerged as a novel and complementary resource for parasitic weed management. The suppressive activity of the soil microbiome was exemplified in the recent study by Kawa et al. [13], in which the microbiome complement of a soil suppressed *Striga* infection of *Sorghum bicolor* by modulating the breakdown of haustorium-inducing factors and induction of root cell wall barriers. Other studies focusing on single microbial taxa, such as the fungus *Fusarium oxysporum*, have shown reduction of *Striga* germination, emergence, and infection rates [14–16]. Collectively, these and other efforts demonstrate the largely unexplored potential of microbes to disrupt the parasite’s life cycle and its interaction with the host plant.

To further disentangle the importance of soil chemistry and the microbiome (i.e. bacterial and fungal community composition) in *Striga* suppression, we profiled 48 Ethiopian field soils from 14 diverse agro-ecological zones for *Striga* infestation levels of sorghum, *Striga* seed bank, soil physico-chemical properties and microbiome composition. We identified the fungal soil community as the most sensitive to variations in the *Striga* seedbank and *Striga* infestation levels monitored in the field. Also, interactions between physico-chemical properties and bacterial and fungal abundance were shown to collectively inform *Striga* prevalence. These data were compiled in a publicly available Shiny App that facilitates multifactorial analyses of fungal, bacterial and physico-chemical properties across the sorghum belt in Ethiopia.

## Results

### *Striga* occurrence and soil physico-chemical properties across Ethiopia

**Fig. 1:**
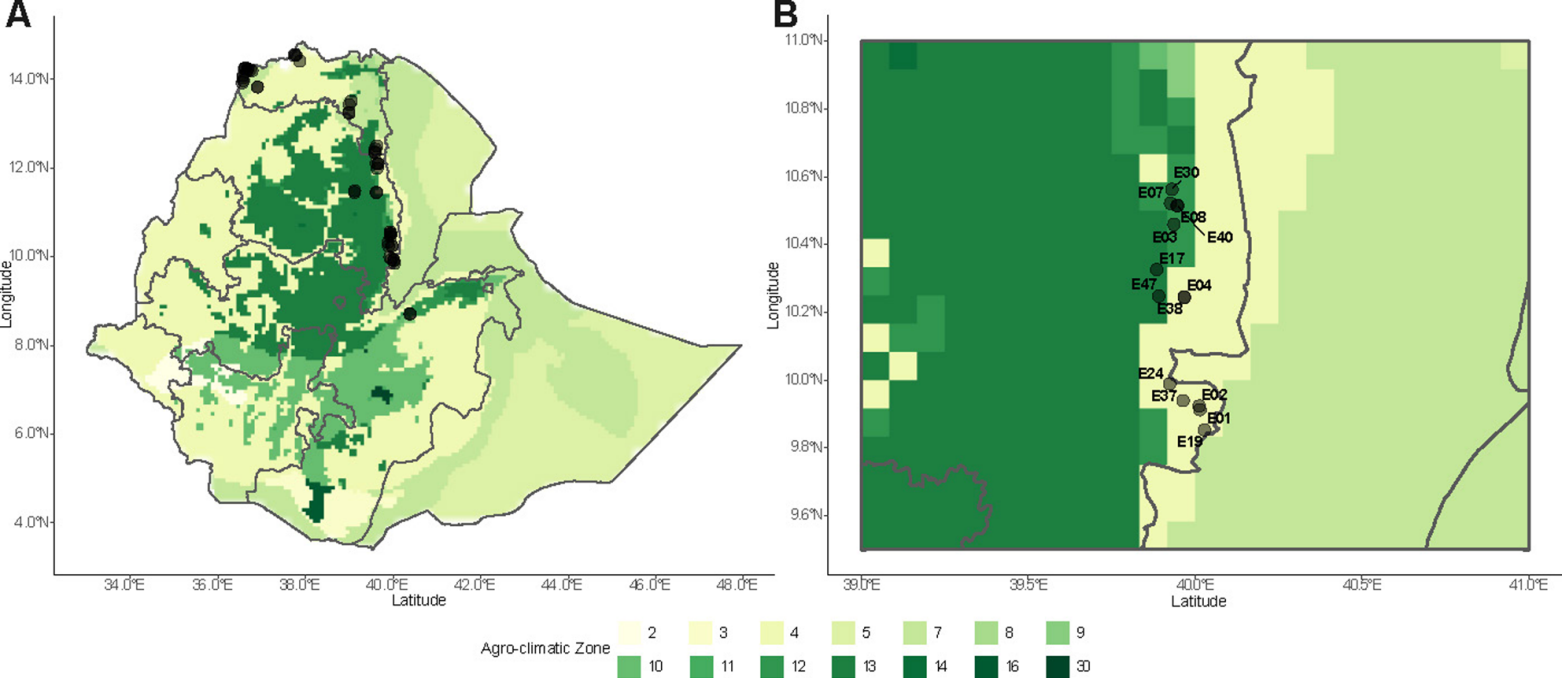
Ethiopian soil samples represent a diversity of agro-climatic zones. A. Map indicating locations of soil samples (black dots) as well as Ethiopian agro-climatic zone (green shades in heatmap) based on the 3-character Koeppen-Geiger climate classification using climatic data from CLUST32 (years 1981-2010) (source: GAEZ v4) (Supp. Data 1). B. Magnification of the Kewet region demonstrating the distance between samples in areas with varying soil and *Striga* infestation and seedbank. Koeppen-Geiger classifications 2- Equatorial monsoon, 3- Equatorial savannah, dry summer, 4- Equatorial savannah, dry winter, 5- Desert climate, hot, 7- Steppe climate, hot, 8- Steppe climate, cold, 9-Temperate/mesothermal climate, fully humid, hot, 10-Temperate/mesothermal climate, fully humid, warm, 11-Temperate/mesothermal climate, fully humid, cold, 12-Temperate/mesothermal climate, dry summer, hot, 13-Temperate/mesothermal climate, dry summer, warm, 14-Temperate/mesothermal climate, dry summer, cold, 16-Temperate/mesothermal climate, dry winter, warm, 30-Tundra climate

**Fig. 2:**
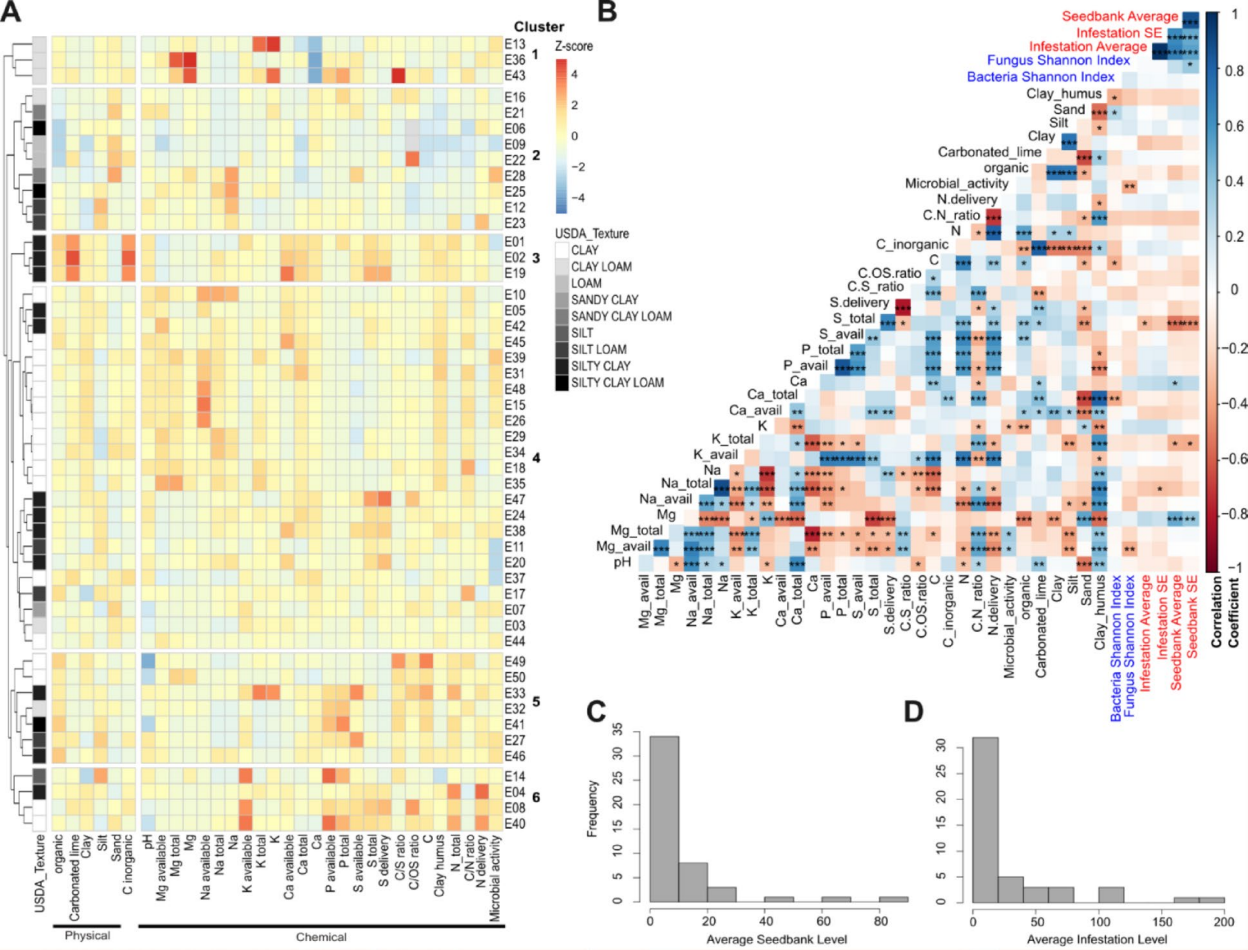
Soil samples vary in *Striga* infestation and seedbank levels, physicochemical parameters and microbial diversity. **A** Heat map of soil physico-chemical properties and *Striga* measurements of all naturally infested soils excluding outlier sample 30. Color represents the relative value across samples (pheatmap, scale = columns). Soil samples were sorted by hierarchical clustering using a Euclidean distance metric with a tree cut off at 6 branches. Row annotations provided for soil texture.** B** Pairwise complete Spearman correlation analysis of soil physico-chemical attributes, microbe diversity, and *Striga* occurrence measurements. Color indicates the correlation value, as calculated by the “psych” package in R. Non adjusted significance levels of 0.05, 0.01, and 0.001 are represented by *, **, and *** respectively. *Striga* parameter labels highlighted in red. **C** Distribution of *Striga* seedbank as seeds per 150 g of soil in soil samples. **D** Distribution of *Striga* infestation as the number of *Striga* in a square meter normalized by the number of sorghum plants

Forty eight soils were sampled across the sorghum belt in Ethiopia to maximize diversity in *Striga* presence, physico-chemical properties, and microbial community composition (Fig. 1) [17]. *Striga* infestation (plants per m^2^, *Striga*/m^2^) and seedbank (*Striga* seeds per 150 g of soil, seeds/150 g soil) varied considerably across the soils and locations, with soils containing no *Striga* infestation (samples E13, E16, E20, E21, E33, E34, E42, and E47) or seedbank (samples E13, E16, E17, E19, E20, E21, E30, E33, E38, E40, E43, and E45) while others presented with maximum infestation of 185.9 *Striga*/m^2^ (sample E12) and 85.5 seeds/150 g soil seedbank (sample E22) (Fig. 2A and B, S1, S2). The Kewet region is notable for its differences in agro-climatic zones and *Striga* occurrence despite close geographical proximity. The thirteen sites in this region were within 80 km of each other and represent four different agro-climatic zones (Fig. 1B). Within the Kewet region, in agro-climatic zone 12, soils E03, E07, E08, E30 and E43 have a highly variable *Striga* seedbank and infestation (25.1, 3.4, 11.9, 0, 0 average seeds/150 g soil and 118, 1.6, 18.4, 0, 10.1 average *Striga*/m^2^, respectively) (Fig. S1). In contrast, soils E01, E02 and E19, spanning an 8 km distance and within the same agro-climatic zone (zone 4), have relatively small seedbank and infestation levels (0.9, 1.9, 0 average seeds/150 g soil, and 0, 0, 1.8 average *Striga*/m^2^) (Fig. S1). Variation in *Striga* infestation relative to the seedbank within similar geographic locations is also present. For example, soils E24 and E37, also in agro-climatic zone 4, are spaced apart by only 7 km and while they have similar seedbank levels (averaging 2.6 and 2.0 seeds/150 g soil, respectively), they differ in infestation values (averaging 68.5 and 28.5 *Striga*/m^2^) (Fig. S1).

Six distinct soil groups were defined based on hierarchical clustering of physical and chemical values (Fig. 2A). Chemical properties drove differences between certain clusters. Cluster 1 is characterized by low collective calcium levels (average 35.3%) and features samples with distinct chemical properties. E13 contains a high potassium content (7.4%, 18.3 mmol K / kg) while E36 had a very high percentage of total magnesium (64%, 156.8 mmol Mg / kg). E43 had a high potassium content (6.4%) and magnesium content (59%), as well as a high carbon-to-sulfur ratio (375). Cluster 3 shows an abundance of carbon (average organic and inorganic 2.9%) and a high available calcium (average 4.5 mmol Ca / L). Physical characteristics also inform the clustering, with Cluster 3 samples characterized by silty clay soil texture heavy in carbonated lime (average 4.9%) and Cluster 2 containing samples that have low levels of clay, clay humus, and organic matter (average 24%, 180 mmol+ / kg, 2.1%). Physical proximity, in some cases, was associated with similar composition. For instance, the physically proximal E01, 02 and 19 had similar levels of calcium, sulfur, inorganic carbon, organic matter and carbonated lime and clustered together in Cluster 3 (Supp. Data 1). Other samples in Kewet; E04, E08 and E40 clustered separately into Cluster 6 and have similar levels of phosphorus, sulfur, potassium and nitrogen. In contrast, the geographically distant sample E14 also clusters in Cluster 6 due to its similar physico-chemical composition to E04, E08 and E40. The largest cluster, Cluster 4, had median levels of physico-chemical properties. Principal Component Analysis (PCA) was used to identify variables that explained the most variation in soil physico-chemical profiles. Together, PC1 and PC2 explained 41.5% of the variation (Fig. S3A). The most influential factors in the physico-chemical PCA were total sodium, available potassium, and available sodium for PC1 and total sulfur, available calcium and total calcium for PC2 (Supp. Data 1).

## Soil properties associated with *Striga* infestation and *Striga* seedbank

We next sought to determine if any soil physico-chemical parameters were significantly correlated with *Striga* infestation. None of the soil texture measurements demonstrated a relationship with *Striga* (Fig. 2B). From overall nutrient composition percentages, calcium (Ca) and magnesium (Mg) positively correlated with average *Striga* seedbank amount (Ca *r* = 0.32, *p* = 0.027; Mg *r* = 0.51, *p* = 0.0002). Meanwhile, negative relationships with *Striga* seedbank were found for total potassium (K, mmol K / kg, *r* = -0.35, *p* = 0.015), and total sulfur content (S, mg S / kg, *r* = -0.54, *p* = 0.00007). Total sulfur content also negatively correlated with average *Striga* infestation (*r* = − 0.31, *p* = 0.03) (Fig. 2B). *Striga* occurrence varied with nutrient levels across sampled soils, suggesting specific nutrients may influence *Striga* prevalence in these regions.

## Bacterial and fungal community composition changes with *Striga* occurrence

The microbiome of these soils was profiled by 16S rRNA and ITS amplicon sequencing, capturing 19,905 and 10,749 unique bacterial and fungal amplicon sequence variants (ASVs), respectively (Supp. Data 2) [18]. Soils had variable bacterial (Fig. S2B) and fungal (Fig. S2C) phyla abundances, dominated by the bacterial phyla Acidobacteria, Actinobacteria and Proteobacteria and the fungal phyla Ascomycota, Basidiomycota, and unidentified phyla. PCA analysis of CLR transformed microbial data demonstrates 9.9% of bacterial community variation explained by PC1 and 8.1% by PC2 (Fig. S3B, Supp. Data 2). The top ASVs driving PC1 were in Acidobacteriota and Proteobacteria phyla (Supp. Data 2). For fungal ASVs, the top 2 PCs explained 15.0% of community variation (Fig. S3C, Supp. Data 2). Of the top 10 ASVs for fungal PC1, ASVs in the Ascomycota phylum, specifically classes Pezizomycetes and Dothideomycetes, were the most informative for variation in fungal community (Supp. Data 2). While informing the PCA, the absolute value of the top ASV loadings of were less than 0.12, suggesting the combination of many taxa most inform the variation of microbial communities seen in the soil samples collected. Alpha diversity of bacterial and fungal communities, as estimated using the Shannon Index, showed little variability in bacterial communities across soils (6.5 ± 0.2), and greater variability in fungal communities (4.6 ± 0.4). (Fig. S1E and H, Supp. Data 2). While the overall level of microbial diversity did not vary dramatically across samples, fungal community diversity positively correlated with the *Striga* seedbank variance (*r* = 0.37, *p* = 0.012) (Fig. 2B, Supp. Data 3). This suggests specific fungal community composition, higher diversity or presence of specific species within that diversity, may associate with *Striga* occurrence.

**Fig. 3:**
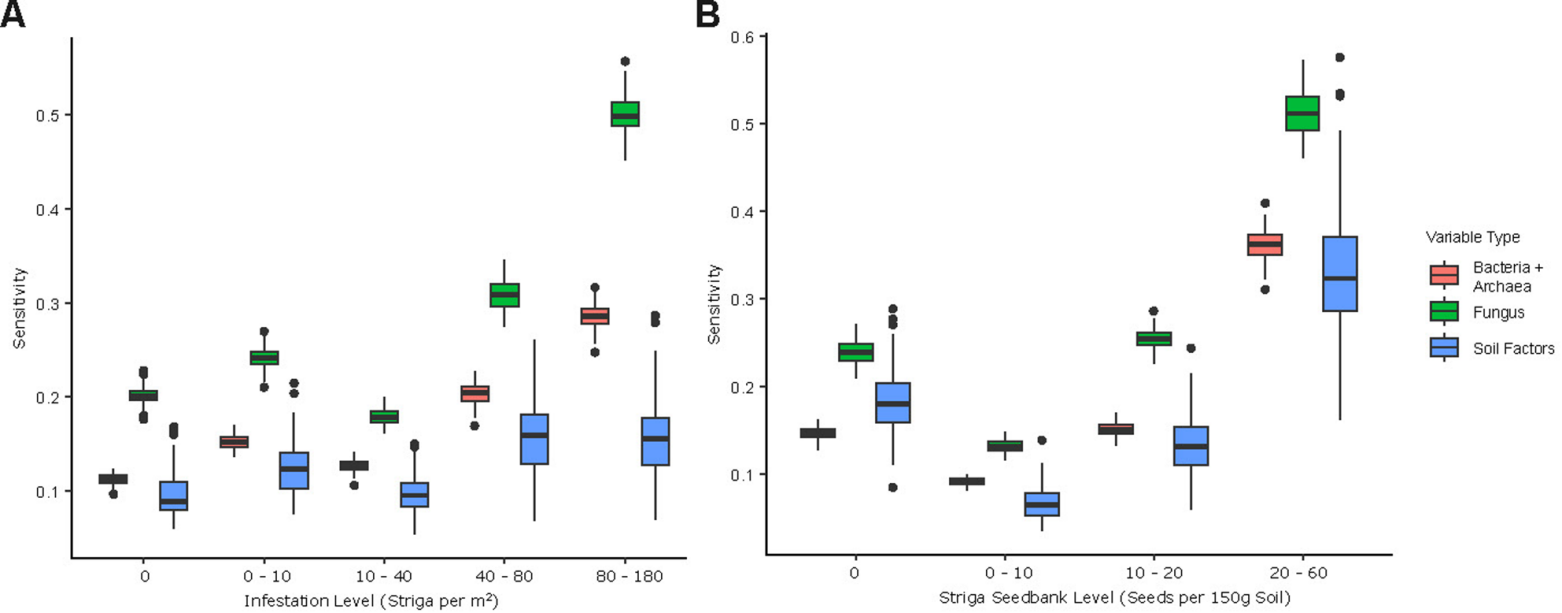
Soil fungal community composition changes the most with varying *Striga* occurrence. (A) Sensitivity analysis of *Striga* field infestation against variable categories (bacteria and archaea, fungi, and soil physico-chemical factors). (B) Sensitivity analysis of the same categories against *Striga* seedbank levels

Generalized Joint Attribute Modeling (GJAM) [19, 20] revealed the sensitivity of microbial groups (bacteria+archaea and fungal communities) to changes in *Striga*. *Striga* infestation and seedbank variables were grouped into four discrete categories to represent zero, low, medium and high levels (Fig. 3). The three different groups (bacteria/archaea, fungi, physico-chemical variables) were submitted to sensitivity analysis with respect to changes in *Striga* infestation (Fig. 3A) and seedbank (Fig. 3B, Supp. Data 3). The fungal community displayed the highest sensitivity values, followed by the bacterial/archaeal community and soil physico-chemical factors. Interestingly, the sensitivity of fungal and bacterial groups (or communities) increases when *Striga* infestation exceeds 40 *Striga*/m^2^ and the *Striga* seedbank surpasses 10–20 seeds/150 g soil. However, for the *Striga* seedbank, we noticed that the microbial communities (fungal and bacterial) and soil physico-chemical attributes still demonstrated relatively high sensitivity values at sites with 0 *Striga* seedbank (Fig. 3B). This suggests that the soils without a *Striga* seedbank harbor a distinct microbiome.

## Specific taxa correlate with *Striga* infestation and seedbank

**Fig. 4:**
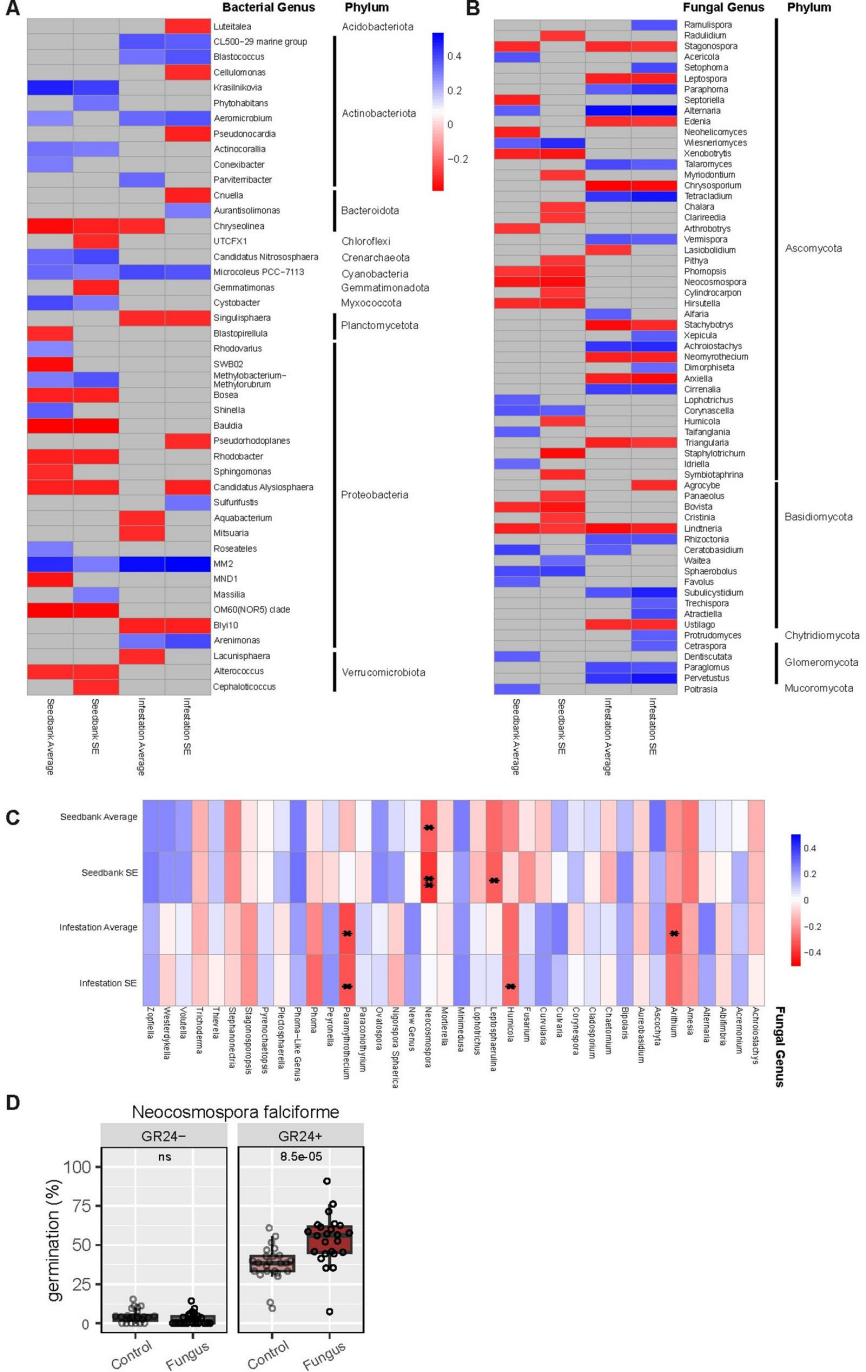
Specific microbial genera negatively correlate with *Striga* occurrence in soils. **A** Bacterial genera with significant correlations to *Striga* occurrence (Pearson, non-adjusted *p*-value < 0.05). **B** Fungal genera with significant correlations to *Striga* occurrence (Pearson, non-adjusted *p*-value < 0.05). Genera of both plots are grouped by phylum for visualization purposes. Color scale is shared for both plots A and B. **C** Pearson correlation results of all reassigned fungal genera with *Striga* occurrence, displayed in alphabetical order. Significance levels of non-adjusted p values 0.05, 0.01, and 0.001 are represented by *, **, and ***, respectively. **D** A strain of the species *Neocosmospora falciforme* was tested for its effect on *Striga* seed germination in vitro with and without the addition of synthetic strigolactone GR24. Statistics presented represent p-values from a Wilcoxon test comparing means within the GR24 treatment

We next determined how specific fungal or bacterial taxonomic groups, from phylum through genus, related to *Striga* infestation, seedbank levels, or their variability by Pearson correlation (Fig. 4A and B, Supp. Data 4). The analyses revealed that correlations with *Striga* shared between nested taxonomic levels were rare. For instance, despite the phylum-level negative correlation of Myxococcota with *Striga* field infestation (*r* = − 0.32, *p* = 0.03), when looking at bacterial composition at the genus level, only one of 17 identified genera demonstrated a significant correlation with *Striga* (Supp. Data 4). *Cystobacter* was the only genus of Myxococcota with significance, yet it showed a positive correlation with seedbank level (*r* = 0.40, *p* = 0.006), demonstrating the extent of variation within a single taxonomic group (Fig. 4A, Supp. Data 4). Bacterial genera: *Lacunisphaera*,* Alterococcus* and *Cephaloticoccus* of the phylum Verrucomicrobiota demonstrated a consistent negative relationship with *Striga* within a phylum (Fig. 4A). *Alterococcus* negatively correlated with seedbank level (*r* = − 0.30, *p* = 0.04), *Cephaloticoccus* negatively correlated with seedbank variance (*r* = − 0.30, *p* = 0.04) and *Lacunisphaera* negatively correlated with *Striga* infestation (*r* = − 0.29, *p* = 0.05) (Supp. Data 4). While these genera were all of the same family, Opitutaceae, the variable abundance of other genera and the unknown ASVs in this taxon was such that an association with *Striga* was lost at the family level. In fact, none of the higher taxonomic groups of this clade; order Opitutales, class Verrucomicrobiae, or phylum Verrucomicrobiota, showed a correlation with *Striga* (Supp. Data 4). Given the variation in microbial life strategies, different associations within a taxonomic level are to be expected.

Both positive and negative relationships were identified between fungal genera and *Striga*. Genera of the Glomeromycota phylum with correlations to *Striga* were each in the positive direction (Fig. 4B). These genera represent two different classes within this phylum, Glomeromycetes and Paraglomeromycetes. In the former, *Cetraspora* correlated with infestation variance (*r* = 0.30, *p* = 0.04) while *Dentiscutata* correlated with seedbank (*r* = 0.31, *p* = 0.038). Of the latter, *Paraglomus* and *Pervetustus* both correlated positively with infestation levels (*r* = 0.34, *p* = 0.022; *r* = 0.38, *p* = 0.009) and its variance (*r* = 0.33, *p* = 0.026; *r* = 0.43, *p* = 0.002) However, neither of these classes correlated with *Striga* on their own (Supp. Data 4). Within the Agaricomycetes class of Basidiomycota are many genera associated with *Striga* occurrence, although in opposing ways, resulting in neither significantly correlating with *Striga*. Genera with negative correlations to *Striga* seedbank were in the Agaricales order; *Agrocybe* (infestation SE *r* = − 0.31, *p* = 0.039), *Panaeolus* (seedbank SE *r* = − 0.29, *p* = 0.049), *Bovista* (seedbank *r* = − 0.31, *p* = 0.036), *Cristinia* (seedbank SE *r* = − 0.29, *p* = 0.047), and *Lindtneria* (seedbank *r* = − 0.33, *p* = 0.027; infestation *r* = − 0.3553, *p* = 0.0154). Even so, the Agaricales order did not show a significant correlation with *Striga*, due to other ASVs in this order with abundance that did not follow this trend (Supp. Data 4). However, order Cantharellales in the Agaricomycetes class positively correlated with seedbank levels (*r* = 0.32, *p* = 0.029) and is bolstered by three of seven identified genera in Cantharellales, also demonstrating positive correlation with *Striga*; *Rhizoctonia* (infestation *r* = 0.32, *p* = 0.030), *Ceratobasidium* (seedbank *r* = 0.35, *p* = 0.016; infestation *r* = 0.30, *p* = 0.044), and *Waitea* (infestation *r* = 0.27, *p* = 0.07). Other genera of the Agariomycetes class also showed positive correlations with various *Striga* measures. *Subulicystidium* and *Trechispora* showed a positive correlation with *Striga* infestation (*r* = 0.40, *p* = 0.006), which coincides with their order, *Trechisporales*, which also showed a positive correlation (*r* = 0.43, *p* = 0.003). Taxonomic groups that demonstrate a consistent negative relationship with *Striga* at multiple levels represent groups of species that may prove fruitful to investigate further for diminishing the *Striga* seedbank or reducing infection.

## Can individuals from potentially suppressive microbial genera directly impact *Striga* seed?

As a proof-of-concept, we aimed to test the capacity of a representative isolate from a microbial genus which was negatively correlated with *Striga* occurrence. To better resolve their taxonomy, ASVs were re-assigned to the genera of individually cultured Ethiopian soil fungal isolates who had the highest bit score match [21] (Supp. Data 5). One of these re-assigned genera was *Neocosmospora* which showed a significantly negative correlation with *Striga* seedbank (*r* = 0.32, *p* = 0.03). A strain of *Neocosmospora falciforme* was tested in vitro for its impact on *Striga* seed viability and germination and demonstrated increased *Striga* seed germination (Fig. 4D, Supp. Data 5). Similarities between the observational and experimental studies suggest that certain taxa, such as *Neocosmospora*, influence the *Striga* seedbank or host infection. Future studies will include testing other fungal and bacterial taxa either alone or in combination (so-called synthetic community, SynCom) for activity against the *Striga* seedbank.

### Provision of data for research and agricultural communities - Shiny app

**Fig. 5:**
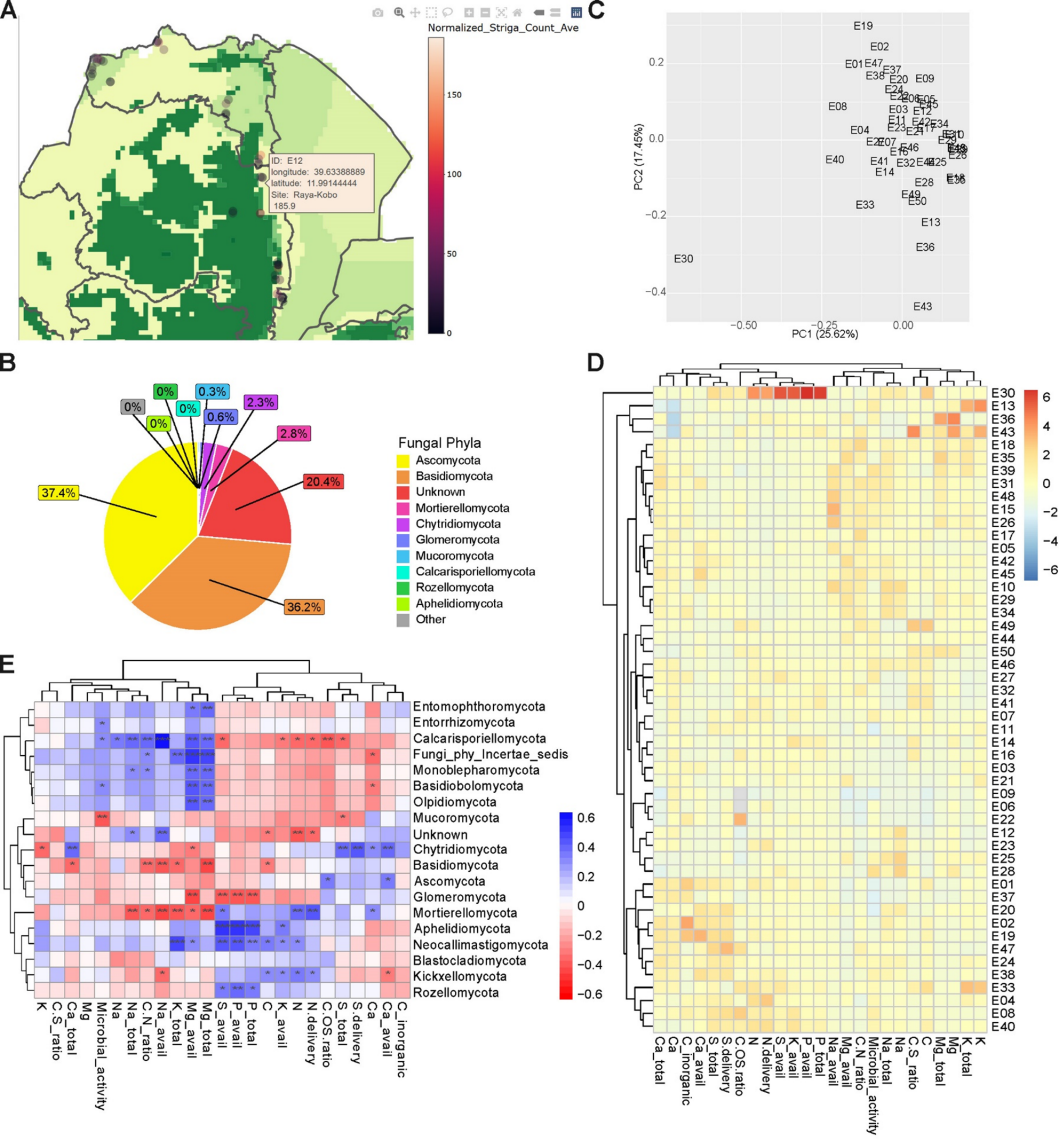
Demonstration of Interactive Application. Users can browse through the soil data using an interactive map (**A**) and get summaries of physio-chemical and microbial compositions for individual sample points (**B**). Data visualization examples of *Striga* infestation level and Fungal phyla composition from soil sample E12 are shown in A & B. Users can explore relationships between samples based on user-selected data in analyses such as PCA (**C**) and generate heatmaps (**D**). In addition to reproducing figures from the paper, users can investigate other correlations between microbial taxa levels and user-selected soil traits (**E**). Plots were saved directly from the app, then altered to fit figure panels here

Analysis strategies from this study, as well as other approaches were integrated into an interactive application (https://brady-lab.shinyapps.io/EthiopianSoil/*).* The purpose of this ShinyApp is to make the data freely accessible and easily analyzed for those working in Sorghum breeding, pest management, microbiology, and soil science in Ethiopia, sub-Saharan Africa, and beyond. This app is equipped with all foundational data described here, including soil physico-chemical and *Striga* measurements, and microbiome ASV counts and taxonomy (Supp. Data 1–3). As an example, a user can explore, in a visually interactive manner, soil physico-chemical and microbial phyla associated with the highest *Striga* field infestation. In the “Map View” window, one can select *Striga* infestation (Normalized_*Striga*_Count_Ave) to be plotted. The color scale indicates the degree of *Striga* infestation, with soil E12 having the highest *Striga* field infestation of 185.9 *Striga*/m^2^ (Fig. 5A, S2, Supp. Data 1). In the “Individual Soil Compositions” window, selecting E12 provides an overview of soil physical and nutrient parameters, as well as microbial composition, broken down by bacterial and fungal phyla (Fig. 5B). All graphs are easily downloadable. One can also perform analyses of multiple samples to determine correlations (using either Pearson or Spearman correlation as a distance metric) with any of the physicochemical, microbial and *Striga* infection parameters. Data can be summarized in a heatmap, used to demonstrate relationships through Pearson and Spearman correlations, lines of best fit, and simplified in a PCA. These strategies allow users to focus on traits or samples most interesting to them based on their own work or prior knowledge. They can have background information for excluding samples from certain areas or only want to look at relationships between certain chemicals. The methods included here can demonstrate patterns or abnormalities to consider in further analysis. For instance, using the app to create a PCA plot for all samples based on chemical analysis data reveals how sample E30 does not cluster regularly with the rest of the soils (Fig. 5C). Observation with the heatmap confirms that E30 is not representative of the other samples due to a six-fold increase from the average in values for phosphorus, potassium, and sulfur measured (Fig. 5D). This sample can easily be removed from the analysis by deselecting it from the sample pull down menu. Microbe data is included in a separate selectable section to walk through data preparation at different taxonomic levels before combining with other user-selected soil data. Correlation analysis can be run at this level to reveal relationships not explored in this text. For instance, a look at fungal phyla correlation with soil chemical measurements revealed phylum Glomeromycota (representative arbuscular mycorrhizal fungi, AMF) shows a negative relationship with available and total phosphorus (*r* = − 0.39, *p* = 0.007; *r* = − 0.38, *p* = 0.008) (Fig. 5E). With these capabilities, users can explore the dataset and download data, analysis and figures, depending on their own research questions and insights. Future efforts will be geared toward improving soil-*Striga* modeling with additional data, enabling it to predict *Striga* pressure and recommend mitigation techniques based on user-inputted information, such as location or soil parameters.

## Discussion

Numerous factors, including the large persistent seedbank and the genetic diversity of *Striga*, play key roles in the distribution and continued devastating effects of *Striga* on crop production in sub-Saharan Africa. This study focused on a smaller, yet highly relevant region within Africa, representing multiple agro-climatic zones and *Striga* variability. Further, we quantified 32 physico-chemical parameters and microbial community composition, representing a diversity of soil composition from farmer’s and other fields under variable *Striga* seedbank and infestation levels in the sorghum belt of Ethiopia. As described previously, assessment of *Striga* presence through soil seedbank quantification is a more reflective measure of actual *Striga* occurrence in operational field sites than recording visible *Striga* plants and is a preferred *Striga* recording strategy [17]. Conversely, qPCR analysis of the seedbank is liable to overestimate *Striga* abundance due to non-viable or degraded *Striga* seeds still present in the soil. Regardless, our data show a significant correlation between the *Striga* seedbank and infestation (*r* = 0.675, *p* < 0.0001), which supports their comparability for assessing *Striga* occurrence [17]. The soil data also reflect high spatial heterogeneity of *Striga* presence within fields, resulting in relatively high standard error measurements for both biological replicates of infestation and technical soil replicates of seedbank. Hence, accurately and comprehensively recording *Striga* seedbanks and infestations in active agricultural regions is a complex task that hinders the monitoring of the effectiveness of existing and new mitigation practices.

Previous continental African and global-scale modeling studies have identified soil properties, such as nitrogen and clay content, as important predictors of *Striga* habitat suitability [23]. These patterns are thought to be partly driven by the fact that host plants increase strigolactone secretion, promoting *Striga* germination, in nutrient-poor soils, particularly under phosphorus (P) or nitrogen (N) limitation typical of low-input farming systems [4, 23]. Therefore, one might expect higher soil nutrient levels to reduce *Striga* infestation [24–27]. However, in our study, we did not observe significant correlations between soil P or N and *Striga* occurrence. Instead, we found that potassium (K), sulfur (S), calcium (Ca), and magnesium (Mg) correlated with infestation (Fig. 2B). This divergence may reflect differences in nutrient ranges of study areas or nonlinear relationships with *Striga*. For example, Bellis et al. [22] found *Striga* habitat suitability peaked at intermediate nitrogen levels (400–1000 mg/kg), while our soils ranged more widely (0–3970 mg/kg), potentially obscuring similar trends when fitted with linear models. Regional studies further support the complexity of these relationships. For example, in the Tigray region of Ethiopia, Gebreslasie et al. [24] observed a negative correlation between *Striga* and available P, but no clear relationship with total N. In Nigeria, Ekeleme et al. [25] reported region-specific variation, with Bauchi State showing *Striga* negatively correlated to clay, exchangeable K, and Ca, aligning with some of our results; while conflicting trends emerged in Kano State, underscoring geographic heterogeneity. Dugje et al. [26] also highlighted how relationships between nutrients and *Striga* vary by region in Nigeria. Nutrient-based variation sustains the need to also consider soil texture, which plays a key role in modulating soil moisture, nutrient retention and microbiological communities [9, 28]. Clay content, specifically, has been repeatedly identified as an important variable in *Striga* ecology. Studies in Nigeria observed negative relationships between clay and *Striga* density [25, 26], while in Ethiopia, positive associations with sand are reported [25]. Complexities in the environmental context, interactions among soil properties and historical land use may explain inconsistent findings across studies and highlight the need for further investigation into how soil dynamics affect *Striga* pressure. The data provided herein also presents a resource for exploring the relationships between soil physico-chemical properties and microbial community patterns in more complex analyses.

While amplicon sequencing is typically not precise enough to capture detect intraspecific diversity within a given genus, it was used to record unknown microbes and to capture broader taxonomic trends. We identified a number of rare or taxonomically unknown microbial taxa whose presence or absence could be impactful on the *Striga*-sorghum interaction. This suggests we may be capturing novel or taxonomically poorly resolved taxa that are contributing strongly to variation in the *Striga* seedbank and infestation. Soils across the area studied demonstrated many of these rare ASVs, which could have been better represented with a larger sample set. Bacterial diversity did not vary greatly across the sampled field soils, but fungal diversity did (Fig. 2B, Supp. Data 3). This observation was supported by the sensitivity analysis, which demonstrated that the fungal community is the most responsive to changes in *Striga* occurrence (Fig. 3A and B). We next determined whether specific fungal and bacterial taxa were correlated with *Striga* and, further, whether any could be associated with lower *Striga* infestation. Several fungal and bacterial genera were found to either positively or negatively correlate with *Striga* infestation and the seedbank. Of note is the phylum Glomeromycota, which houses arbuscular mycorrhizal fungi, known for their beneficial interactions with plants under nutrient-poor conditions. This phylum negatively correlated with levels of phosphorus in the soil, while genera within this taxon positively correlated with *Striga* (Figs. 4B and 5E, Supp. Data 4). It should be noted that ITS amplicon sequencing with universal fungal primers may underestimate AMF diversity and relative abundance, as Glomeromycota have relatively few ITS copies and high intra-isolate ITS sequence variability. Targeted AMF-specific amplicon approaches (e.g., SSU rRNA-based markers) would provide more comprehensive resolution of the mycorrhizal community response to *Striga* occurrence and should be considered in future studies.

Targeted analysis of a strain belonging to the genus *Neocosmospora* validated the hypothesis that a fungal isolate can increase *Striga* germination, indicative of the potential of this fungal genus to induce suicidal germination (Fig. 4D). Promotion of germination under unsuitable conditions in absence of the host, also referred to as suicidal germination, is a highly promising strategy to diminish the *Striga* seedbank over time [22]. Although this proof-of-concept using *Neocosmospora* demonstrates that specific fungal taxa can significantly impact *Striga* seed germination, this effect may only apply to certain species or even strains within the genus. It is also noted that in vitro settings may not recapitulate the microbial dynamics and activities in complex conditions that exist in the field, and therefore, it is likely to also find mismatches between the results of our observational and experimental approaches. Therefore, caution is required when inferring causal relationships from correlative microbiome data. Efforts to understand the links between microbial communities and plant interactions will be continued and pursued collaboratively to generate large-scale data on the functional potential of soil fungi and bacteria to interfere, either alone or in consortia, with the life-cycle of *Striga* and other devastating root-parasitic weeds. Functional validation would preferably encompass a multifactorial experimental design that also includes several potential abiotic drivers (K, S, Ca, and Mg) of *Striga* occurrence.

## Conclusions

Using 48 soil samples from sites with varying levels of *Striga* field occurrence across Ethiopia, we evaluated the roles of soil physico-chemical properties and the microbiome in *Striga* infection. Specific soil parameters were shown to vary with *Striga* occurrence. Calcium and magnesium nutrient profile content positively correlated with *Striga* seedbank, while potassium and sulfur negatively correlated with *Striga* infestation and seedbank levels. The microbiome of these soils was profiled, and while both fungal and bacterial genera showed correlations with measures of *Striga* field occurrence, fungal communities were more responsive than bacteria to changes in *Striga* infestation and seedbank. Collectively, these demonstrate the complexity of the soil-microbiome-*Striga* relationship and the potential of multiple additional modes of control for this devastating parasitic weed. We further validate our findings with one soil isolate from the fungal genus *Neocosmospora*, which negatively correlated with the *Striga* seedbank and promoted *Striga* germination in vitro. This demonstrates the ability of an observational study to identify candidate native microbes that could influence *Striga* by altering germination, suggesting the potential for biocontrol. Research on the interactions between *Striga* and their host species within sub-Saharan Africa is often limited for economic and other reasons. We share these physicochemical and microbial data in a publicly available Shiny app to facilitate continued research on methods for *Striga* control.

### Methods

#### Soil collection

A total of 50 soil samples were taken from naturally and artificially *Striga* infested fields in Amhara (Kemise, North Shewa, South and North Wollo Zones) and Tigray (West, Central and South zones) regions of Ethiopia in October, 2017 as described within Mitiku et al. [17]. Forty-eight soil samples were collected from naturally infested agro-ecological zones as well as two from artificially infested fields (Fig. 1). Sorghum fields with four categories (zero, low, medium and high) of *Striga* field infestation were randomly selected, and 4 samples were taken from the top layer (0–20 cm) around the root zone of a sorghum plant and then combined to form one composite sample per field. The exceptions were samples E10, E15, E42, E44, E45, & E48, where sorghum plants were not uniformly distributed and only 1 sample was taken. *Striga* infestation and the *Striga* seedbank were quantified for forty-eight naturally infested soils (Fig. S1 and 2C–D). Physico-chemical parameters were determined from forty-eight soils (Fig. 2A, S2A).

### Measurement of *Striga* infestation and seedbank

*Striga* emergence was counted from four randomly chosen spots per field site, and the number of sorghum plants counted per m^2^ was used to normalize the number of emerged *Striga* plants. *Striga* emergence data were not obtained for samples E15 and E48 because these were from artificially *Striga*-infested fields. Seed bank density was assessed in Mitiku et al. [17] using a qPCR approach.

### Soil physical and chemical parameters

Soil physico-chemical analysis was done at Eurofins Agro (Eurofins Agro, Wageningen, The Netherlands) (Supp. Data 1) [18] The 4 macronutrients required in the highest amount by plants (N, K, Ca, Mg) were also described in percentages of nutrient make-up. H and Al were also measured for this purpose, but all samples were below the minimum detection threshold and discarded from further analysis. Datasets used in physico-chemical and *Striga* analyses excluded artificially infected fields (Samples E15 & E48) as well as an outlier sample, E30.

### Microbial sequencing and processing

The composite bacterial and fungal microbiome were quantified for forty-six soils, excluding two soils which had been planted a separate experiment involving intercropping systems (E49 and E50) (Fig. S2 B and C). Bacterial DNA preparation, sequencing, and processing are explained in Abera et al. [18]. The same DNA samples were used for fungal sequencing targeting the ITS2 region of Internal Transcribed Spacer (ITS) gene, using primers ITS3 (5′- GCATCG ATGAAGAACGC-3′) and ITS4 ( 5′- TCCTCCGCTTATTGATATGC-3′) identified in White et al. [29]. The PCR conditions were an initial denaturation step at 96 °C for 15 min, followed by 33 cycles of 96 °C for 30 s, 52 °C for 30 s, 72 °C for 60 s, and a final extension step at 72 °C for 10 min. The PCR mix contained 1 µL of DNA template, 1× of Qiagen 10× Buffer with 15 mM MgCl2, 5% Roche DMSO, 0.2 mM dNTP mix 10 mM NEB, 0.02 U µL − 1 Qiagen HotStar Taq 5 U µL − 1, 0.6 µM of the ITS3, 0.6 µM of the ITS4 and sterile Milli-Q water up to the final volume of 25 µL. For negative controls, 1 µL sterile water was added in place of DNA. Verification of amplification was performed on 2% agarose gel. The amplicons were sequenced with Illumina MiSeq by BaseClear (Leiden, Netherlands). Paired-end Illumina reads were processed using the DADA2 pipeline (v1.16) in R [30]. Raw read sequences containing ambiguous bases were removed. Primer sequences were removed using cutadapt. Quality filtering was based on expected errors, with a maximum of 2 expected errors allowed per read for both forward and reverse reads. Error models were learned separately for forward and reverse reads, and amplicon sequence variants (ASVs) were inferred using DADA2’s denoising algorithm. Forward and reverse reads were merged, and a sequence table was constructed. Chimeric sequences were identified and removed using the consensus method, retaining approximately 97% of total reads. Taxonomic assignment of non-chimeric ASVs was performed using the Silva 138.1 prokaryotic SSU taxonomic training data and the UNITE fungal reference database (general release, dynamic version 10.05.2021) [31, 32]. Final outputs included an ASV abundance table, taxonomic assignments, and representative sequences, formatted for downstream ecological and statistical analyses. Some samples were sequenced twice (E05, E33, E36 and E48), for these, ASV counts were averaged for later analysis. ASV data was rarefied (Rarefaction curves in Supp. Fig. S1) and alpha diversity measures (Observed, Chao1, ACE, Shannon, Simpson and Fisher) were calculated using phyloseq (1.46.0). Data used in community abundance and correlation analyses were zero-imputed and CLR transformed. PCA analysis was then conducted using R base package “stats” (4.3.1), an appropriate approach on CLR transformed data as stated in Gloor et al. [33].

### Generalized joint attribute modeling (GJAM)

To integrate microbiome data with *Striga* infection and soil attributes, we used generalized joint attribute modelling (GJAM) via gjam package version 2.6.2 in R v4.3.1 [19]. To evaluate the role of *Striga* infection and *Striga* seedbank on the microbial communities (bacteria and fungi) we grouped those variables into discrete categories to represent Zero, Low, Medium, and High levels of both *Striga* infestation and *Striga* seedbank. Then two models were generated to evaluate (1) the effects of *Striga* Infestation and (2) the effects of *Striga* Seedbank (Supp. Data 3). GJAM is a Bayesian model that estimates the regression coefficient via Gibbs sampling. In our case, we used 20,000 iterations before reaching stability of the regression coefficients. Composition count was selected as data type since the microbiome data is to be considered compositional based on heterogeneous number of reads per sample (Gloor et al.) [33]. The impact of the categories of *Striga* Infestation (model 1) and *Striga* Seedbank (model 2) in shaping the microbiome and soil attributes was evaluated per groups of variables via Sensitivity analysis as in Rotoni et al. [34]. The sensitivity to predictors analysis evaluated how the different explanatory variables (in our case the different levels of *Striga* seedbank and infestation) influenced the different groups of variables, thus allowing us to identify the levels of *Striga* seedbank and infestation that resulted in the biggest shifts in the microbial community and soil attributes.

### Correlation analyses

*Striga* data followed imputation with a constant value of 1 then log 10 transformations to be used in Pearson and Spearman correlations against physico-chemical and microbial soil data using the R package “psych” (2.4.3). Soil samples missing measurement categories (i.e. E15 and E48 which did not have *Striga* measurements, E49 and E50 which did not have microbial data) were excluded from analyses requiring that data. E30 was excluded from all physico-chemical related analyses for being an outlier. Soil texture percentage traits (Clay, Silt, Sand, Carbonated lime, and Organic) and nutrient percent composition traits (Carbon, Magnesium, Sodium, Potassium, and Calcium) were normalized to 100% and clr transformed separately before correlation analyses was run. Bonferroni p-value adjustment was applied to results (Supp. Data 3–4).

### Fungal genera reassignment

Fungal isolates from Lombard et al. [35] and Maciá-Vicente et al. (unpublished) were collected from the same soils analyzed in this study. Fungal ASV sequences were aligned with the sequences from isolates of these soils deposited in GenBank [accession numbers PX349626 - PX351653] using nucleotide BLAST+ [21, 35–37]. Matches were filtered to the highest bit score. The ASVs were then reassigned the genera of their best match isolate. In the event an ASV sequence matched multiple accessions, the ASV was assigned the genera that represented at least 70% of its matches (Supp. Data 5). Microbial data followed summation at different taxa levels, zero imputation with a constant value and centered log-ratio transformation from package Tjazi (0.1.0.0) using the “logunif” method at 1000 repetitions [38].

#### In vitro *Striga* assays

Sterilized *Striga* seeds collected from Qilee in 2022 were imbibed in 1% water agar and incubated 6 days at 30 C in the dark. Seeds were treated with the following: no fungus & no GR24+, fungus & no GR24, no fungus & GR24, fungus & GR24. Fungi were inoculated by applying a suspension of 1000 conidia/replicate, after which plates were incubated at 30 C in the dark for 6 days. Seed were then treated/untreated with 1 mg/ml GR24, and after 24 h the percentage of seed germination per treatment scored (Supp. Data 5). Treatments consisted of 24 replicates, each with 20–80 seeds.

### Interactive application

The web application was made using the shiny package (1.8.0) in R. It contains all the soil data included in this article (physico-chemical soil analysis, agro-climatic zones, summary *Striga* measurements, and ASV sequencing results) (Supp. Data 1–2). The app was equipped with similar analyses as presented in this study with additional ways of visualizing the data. Maps in the paper and app were created using packages “ggplot2” (3.5.1), “plotly” (4.10.4) and “sf” (1.0–15) and polygons from the github repository of user georgique and https://gadm.org/. Correlation analyses followed R package “psych” (2.4.3), PCA analysis used the base “stats” (4.3.1) package, and scatter plots were produced using base “stats” and graphics (4.3.1). Random forest and decision tree analysis (randomForest 4.7–1.2) was included for demonstrative purposes of future use, using all selected samples in the training dataset and was not equipped for a testing dataset to assess accuracy. Scripts for this ShinyApp can be found in the following github repository: https://github.com/TameraTaylor/EMIC-Ethiopian-Soil-Analysis.

## Supplementary Information

Below is the link to the electronic supplementary material.

Supplementary Data 4

Supplementary Data 5

Supplementary Data 1

Supplementary Data 3

Supplementary Data 2

Supplementary Figures

Supplementary Data List

## Data Availability

All data generated during this study are included in the supplementary information files or available in public repositories. Raw ASV sequence reads are available on NCBI at the BioProject number PRJNA841233 (https://www.ncbi.nlm.nih.gov/bioproject/?term=PRJNA841233) and ENA at study ID PRJEB104932 (https://www.ebi.ac.uk/ena/browser/view/PRJEB104932). Sequences for deeper taxonomic resolution are from GenBank [accession numbers PX349626 - PX351653] (https://www.ncbi.nlm.nih.gov/genbank/). Scripts used in analysis are available at https://github.com/taffymieh/Ethiopian-Soil-Analysis.

## Abbreviations

(*Striga*): *Striga hermonthica*
(Sorghum): *Sorghum bicolor*
(AMF): Arbuscular mycorrhizal fungi
(*Striga*/m^2^): *Striga* plants per m^2^
(seeds/150 g soil): *Striga* seeds per 150 g of soil
(Supp.): Supplemental
(SE): Standard Error
(PCA): Principal Component Analysis
(K): Potassium
(Ca): Calcium
(S): Sulfur
(Mg): Magnesium
(GJAM): Generalized Joint Attribute Modeling
