# Supplementary material for: Disentangling the importance of microbiological and physico-chemical properties of Ethiopian field soils for the Striga seed bank and sorghum infestation
Source: Environ Microbiome. 2026 Jul 13;21:91. doi: 10.1186/s40793-026-00926-3 (PMC13397753; doi:10.1186/s40793-026-00926-3)
Supplement: Supplementary file 6 — Supplementary Figures [file 40793_2026_926_MOESM6_ESM.pdf]

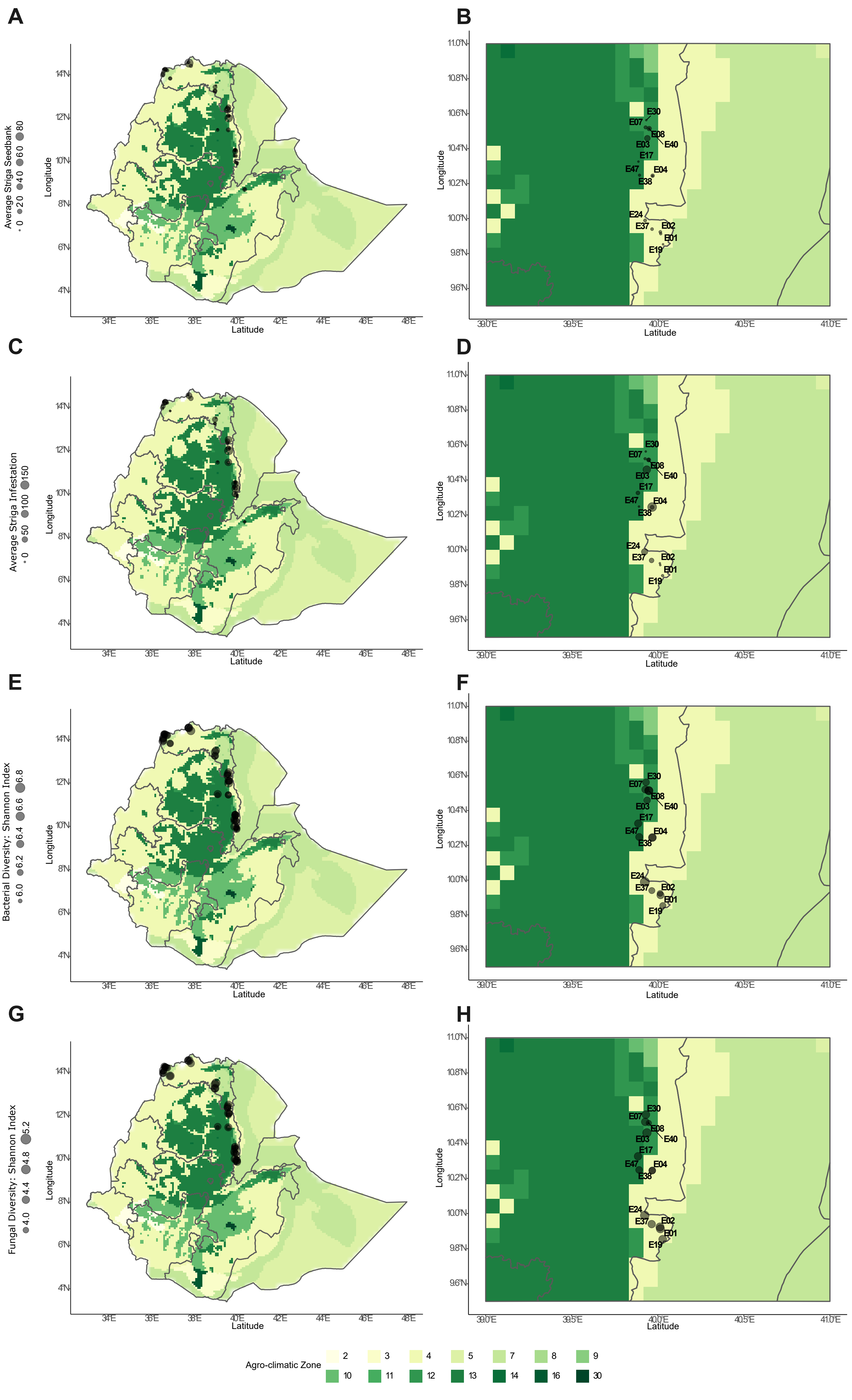

S Fig1. Striga occurrence and microbiological diversity vary across soil samples.
Striga seedbank and field infestation levels varied across the sampling sites **(A & C)**, even between sites of close proximity such as in the Kewet area **(B & D)**. The shannon index, a metric of alpha diversity was calculated separately for fungal and bacterial microbes and also varied between sites **(E-H).**

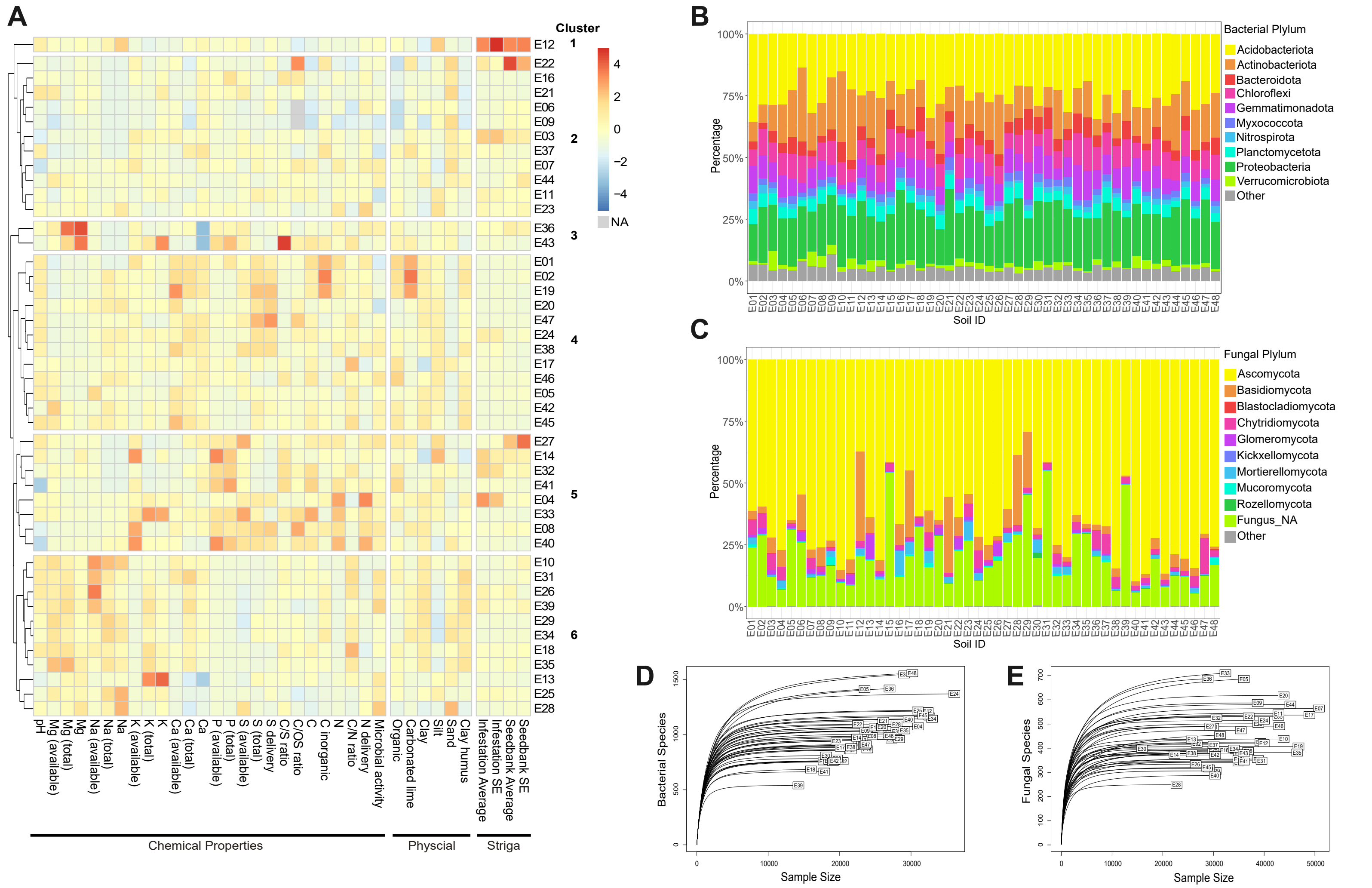

S Fig2. Some soil factors negatively correlate with Striga seedbank or field infestation.

A. Heat map of soil physico-chemical properties and Striga measurements of all naturally infested soils excluding outlier sample 30. Color represents the relative value across samples (pheatmap). Soil samples were sorted by hierarchical clustering using a Euclidean distance metric with a tree cut off at 6 branches. **B.** Proportions of the most abundant bacterial phyla within each soil sample. **C.** Proportions of the most abundant fungal phyla within each soil sample. **D.** Rarefaction curves of bacterial species in all soil samples. **E.** Rarefaction curves of fungal species across all soil samples.

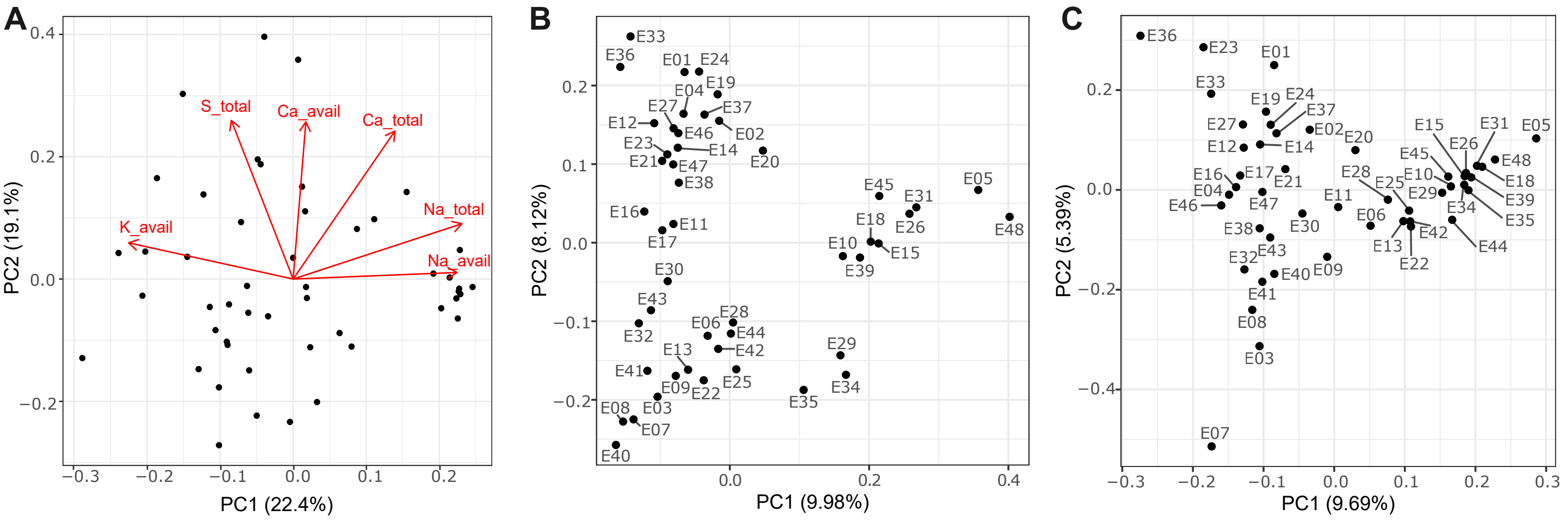

S Fig3. Soil sample variability is driven by many factors.

A. PCA (Principle Component Analysis) of physico-chemical composition. Red arrows represent loading vectors of the 3 most informative factors driving variation in each PC1 (Na_total, K_avail, Na_avail) and PC2 (S_total, Ca_avail, Ca_total) for the physico-chemical composition. **B.** PCA of CLR transformed bacterial ASV community of Ethiopian soil samples. **C.** PCA of CLR transformed fungal ASV community of Ethiopian soil samples.
