# Supplementary material for: Disentangling the importance of microbiological and physico-chemical properties of Ethiopian field soils for the Striga seed bank and sorghum infestation
Source: Environ Microbiome. 2026 Jul 13;21:91. doi: 10.1186/s40793-026-00926-3 (PMC13397753; doi:10.1186/s40793-026-00926-3)
Supplement: Supplementary file 7 — Supplementary Data List [file 40793_2026_926_MOESM7_ESM.docx]

**Supplemental Data 1.xlsx**

Soil geographical, agro-climatic, physico-chemical, and summarized microbial data.

1. Physico-chemical: Soil identifier (ID); location (site); Altitude (meters); latitude (degrees); longitude (degrees); pH; soil sample chemical analysis of total (_total) and available (_avail) elements, N (nitrogen), S (sulfur), P (phosphorus), K (potassium), Ca (calcium), Mg (magnesium); carbon (C, %); soil sample physical analysis (%) of organic matter (organic), inorganic carbon (C_inorganic), carbonated lime (carbonated_lime), clay (<2 um), silt (2-50 um), and sand (>50 um); soil nutrient profiling (%) of calcium (Ca), magnesium (Mg), potassium (K), sodium (Na); nitrogen delivery (N.delivery); sulfur delivery (S.delivery); carbon: nitrogen ration (C/N_ratio); carbon: sulfur ratio (C/S_ratio); hydrocarbon concentration between original and tested soil (C/OS_ratio); electrical exchange measurements of Cation Exchange Capacity (Clay_humus, mmol+/kg); microbial activity (mg N/kg); USDA soil texture (Soil_Texture); and Koeppen-Geiger climate type (Agro_Climate_type).
2. Striga Measurements: Pooled Striga measurements of seedbank and field infestation and their log (base 10) transformations used in analysis.
3. Field Infestation: original Striga field infestation measurements normalized by sorghum presence.
4. Seedbank: Original Striga seedbank measurements by qPCR (Striga seeds per 150g).
5. Land Use: Information about cropping history obtained for soil sample locations.
6. PCA Summary: Importance of components from physico-chemical trait PCA analysis. Excluded sample E30 (outlier) and variable C/OS_ratio (missing values).
7. PCA Eigens Physico-chemical: PCA eigen values for variables in physico-chemical analysis obtained by extracting the loadings from the PCA result of prcomp(). PCs 1-30 to explain 100% of the variation.

**Supplemental Data 2.xlsx**

Microbe sequencing data.

1. 16S Sequencing ASV counts: Bacterial and Archea ASV counts by 16S sequencing with technical replicates (samples E5, E33, E36, and E48) averaged.
2. ITS Sequencing ASV Counts: Fungal ASV counts by Internal Transcribed Spacer sequencing with technical replicates (samples E5, E33, E36, and E48) averaged.
3. Bacteria Diversity: Diversity indexes calculated on rarefied ASVs by the phyloseq package.
4. Fungus Diversity: Diversity indexes calculated on rarefied ASVs by the phyloseq package.
5. PCA Importance: Importance of components from Bacterial and Fungal PCA analysis.
6. Bacteria Loadings: PCA loadings for ASVs and their taxonomy.
7. Fungal Loadings: PCA loadings for ASVs and their taxonomy.

**Supplemental Data 3.xlsx**

Summary correlation analyses.

1. GJAM Infestation: Sensitivity values from GJAM analysis for soil measurements grouped as bacteria, fungus, soil attributes, and Striga calculated for ranges of Striga infestation.
2. GJAM Seedbank: Sensitivity values from GJAM analysis for soil measurements grouped as bacteria, fungus, soil attributes, and Striga calculated for ranges of Striga seedbank.
3. Spearman R values: R value results of all pairwise Pearson correlation analysis on physico-chemical, microbe diversity and log (base 10) transformations of Striga seedbank (average and SE) and infestation (average and SE).
4. Spearman P values: Significance of all pairwise Pearson correlation analysis on physico-chemical, microbe diversity and log (base 10) transformations of Striga seedbank (average and SE) and infestation (average and SE). P value in bottom triangle, Bonferroni adjusted p values in top triangle.

**Supplemental Data 4.xlsx**

Taxon level correlation analysis.

ASV data was aggregated at each taxon level, imputed and CLR transformed. Unidentified taxa were grouped into an "Unknown". Pearson correlation was run with each dataset against log (base 10) transformations of Striga seedbank (average and SE) and infestation (average and SE). Structure of each sheet is the same. Contains R value, P value, and Bonferroni adjusted P value.

1. Bacterial Phyla
2. Bacterial Class
3. Bacterial Order
4. Bacteria Family
5. Bacterial Genera
6. Fungal Phyla
7. Fungal Class
8. Fungal Order
9. Fungal Family
10. Fungal Genera

**Supplemental Data 5.xlsx**

Reassigned fungal genera and in vitro assay.

1. ASV Accession Matches: Best sequence match results between ASV sequences and GenBank accession records of fungal cultures isolated from these same soils.
2. New Fungal Genera Correlation: Pearson correlation analysis results on Fungus genera based on taxonomy from matching ITS sequence in fungal strain collection and log (base 10) transformations of Striga seedbank (average and SE) and infestation (average and SE). Contains R value, P value, and Bonferroni adjusted P value.
3. Assay Data: Treatments and Striga germination measurements from testing fungal strain interaction with striga seeds.
